# Supplementary material for: Dyslipidemia, Obesity, and Ethnicity in Mexican Children
Source: Int J Environ Res Public Health. 2021 Dec 1;18(23):12659. doi: 10.3390/ijerph182312659 (PMC8656470; doi:10.3390/ijerph182312659)
Supplement: Supplementary file 1 [file ijerph-18-12659-s001.zip › ijerph-1444388-supplementary.pdf]

Table S1. **The ability of body fat percentage to discriminate lipid disorder**. Areas under the ROC curve (AUCs), 95% confidence intervals and p-values (P) for cardiometabolic risk factors in school children from Mexico City (Central Mexico).

| | AUC (95%CI), P | $P^1$ |
|---|---|---|
| High TG | 0.710(0.65-0.76), < 0.0001 | 0.062 |
| High TC | 0.57(0.51-0.63), 0.017 | 0.022 |
| Low HDL | 0.70(0.63-0.77), < 0.0001 | 0.002 |
| High LDL | 0.61(0.55-0.68), 0.001 | 0.113 |
| Low ApoA1 | 0.51(0.40-0.62), 0.870 | 0.065 |
| High ApoB | 0.64(0.57-0.69), 0.010 | 0.008 |
| Dyslipidemia | 0.68(0.61-0.75), 0.0001 | 0.734 |

Triglycerides (TG, mg/dL), Total cholesterol (TC, mg/dL), high density lipoprotein, (HDL in mg/dl), low density lipoprotein, (LDL in mg/dl), apolipoprotein A1, (ApoaA, mg/dl), apolipoprotein B, (ApoB mg/dl). Lipids levels classification were according to the borderline-high criteria of the Expert Panel on Integrated Guidelines for Cardiovascular Health and Risk Reduction in Children and Adolescents National Heart, Lung and Blood Institute. P-value ($P^1$ ) resulted from Z test from de body mass index and BFP AUCs pairwise comparisons.

Table S2. **Results of lipid disorder association analysis with obesity.** Obesity (OB), Body Fat Percentage (BFP) school children from Mexico City. Estimate, standard errors (SE), p-value (p), odd ratio (OR) and 95% confidence intervals and p-values (P).

| **HighTG** | **Estimate** | **z** | **OR** | **95%CI** | **P** |
|---|---|---|---|---|---|
| Int | -0.014 | -0.085 | | | 0.930 |
| BFP-OB | 1.329 | 5.405 | 3.78 | (2.34-6.16) | <0.001 |
| **High TC** | | | | | |
| Intercept | -0.158 | -0.932 | | | 0.350 |
| BFP-OB | 0.555 | 2.46 | 1.74 | (1.12-2.71) | 0.014 |
| **Low HDL** | | | | | |
| Intercept | -3.319 | -5.752 | | | <0.001 |
| BFP-OB | 1.353 | 3.796 | 3.4 | (1.77-6.98) | 0.001 |
| **High LDL** | | | | | |
| Intercept | -1.517 | -6.871 | | | <0.001 |
| BFP-OB | 0.871 | 3.243 | 2.39 | (1.42- 4.10) | 0.001 |
| **Low APOA1** | | | | | |
| Intercept) | 2.359 | 7.812 | | | <0.001 |
| BFP-OB | 0.021 | 0.054 | 1.02 | (0.46- 2.22) | 0.960 |
| **High APOB** | | | | | |
| Intercept | -1.089 | -5.573 | | | <0.001 |
| BFP-OB | 0.844 | 3.457 | 2.32 | (1.45-3.79) | <0.0001 |
| **Dyslipidemia** | | | | | |
| Intercept | 1.862 | 3.181 | | | 0.002 |
| Age | -0.119 | -1.874 | 0.88 | (0.78-1.00) | 0.060 |
| BFP-OB | 1.438 | 4.6 | 4.21 | (2.31-7.92) | <0.001 |

Lipids levels classification were according to the borderline-high criteria of the Expert Panel on Integrated Guidelines for Cardiovascular Health and Risk Reduction in Children and Adolescents National Heart, Lung and Blood Institute.
